# Inside out: optimization of lipid nanoparticle formulations for exterior complexation and in vivo delivery of saRNA

**DOI:** 10.1038/s41434-019-0095-2

**Published:** 2019-07-12

**Authors:** Anna K. Blakney, Paul F. McKay, Bárbara Ibarzo Yus, Yoann Aldon, Robin J. Shattock

**Affiliations:** Department of Medicine, Imperial College London, London, UK

**Keywords:** Gene delivery, RNA vaccines, Nanoparticles

## Abstract

Self-amplifying RNA (saRNA) is a promising biotherapeutic tool that has been used as a vaccine against both infectious diseases and cancer. saRNA has been shown to induce protein expression for up to 60 days and elicit immune responses with lower dosing than messenger RNA (mRNA). Because saRNA is a large (~9500 nt), negatively charged molecule, it requires a delivery vehicle for efficient cellular uptake and degradation protection. Lipid nanoparticles (LNPs) have been widely used for RNA formulations, where the prevailing paradigm is to encapsulate RNA within the particle, including the first FDA-approved small-interfering siRNA therapy. Here, we compared LNP formulations with cationic and ionizable lipids with saRNA either on the interior or exterior of the particle. We show that LNPs formulated with cationic lipids protect saRNA from RNAse degradation, even when it is adsorbed to the surface. Furthermore, cationic LNPs deliver saRNA equivalently to particles formulated with saRNA encapsulated in an ionizable lipid particle, both in vitro and in vivo. Finally, we show that cationic and ionizable LNP formulations induce equivalent antibodies against HIV-1 Env gp140 as a model antigen. These studies establish formulating saRNA on the surface of cationic LNPs as an alternative to the paradigm of encapsulating RNA.

## Introduction

Biotherapeutics based on messenger RNA (mRNA) are a promising strategy for both vaccines and protein replacement therapy. To date, mRNA has been used preclinically for a variety of vaccine indications, including infectious diseases such as influenza [1, 2], rabies virus [3], RSV [4], HIV-1 [5, 6], HCV [7], Zika virus [8], and Ebola virus [9], as well as for cancer vaccines, including lung cancer [10], prostate cancer [11], pancreatic cancer [12], and melanoma [13]. Furthermore, a number of mRNA vaccines against both infectious disease and cancer indications are currently being tested in various human clinical vaccine trials at different stages [14]. Self-amplifying mRNA (saRNA) is an alternative approach to mRNA vaccines, and has been shown to induce immune responses with lower doses of saRNA than mRNA (10- to 100-fold lower) [15], and results in prolonged protein expression for up to 60 days in vivo [4]. Upon delivery into the cytoplasm, an saRNA is translated to produce four nonstructural proteins that make up the replicase, which is able to make multiple identical copies of the original strand of RNA, resulting in exponentially more copies of RNA [16]. Whether mRNA (2000–5000 nt) or saRNA (8000–10,000 nt) is used for gene delivery, it is necessary to pair it with a delivery platform in order to enhance cellular uptake.

mRNA has been previously formulated using a range of cationic delivery platforms, wherein the overarching principle is to use a cationic/ionizable lipid or polymer to electrostatically complex the anionic RNA molecules, reducing the size of the particle and facilitating cellular uptake. Approaches have explored the use of polyplexes [11, 17], emulsions [5, 18], and lipid nanoparticles (LNPs) [19, 20]. Currently, the only FDA-approved RNA-based therapy is patisiran, a small-interfering RNA (siRNA)-based therapy for the treatment of hATTR amyloidosis, which is formulated in liposomes prepared using an ionizable lipid [21]. In order to enhance mRNA delivery efficiency, extensive optimization of LNP formulations has been performed, including in vivo design of experiment approaches, which have been shown to enhance the mRNA efficacy just by optimizing the formulation components. However, these specific formulations do not necessarily enhance the efficacy of other types of RNA, such as siRNA [22].

Previous studies with LNP formulations have shown that encapsulating RNA within the particle protects the RNA from degradation by RNAse compared with naked RNA [4]. However, to our knowledge, previous studies have not explored whether RNA needs to be encapsulated within the LNP in order to protect it, or whether complexation to the surface of the particle is sufficient for protection and/or delivery. Logically, cationic LNPs should be able to complex RNA in a similar manner to polyplexes, which have been shown to protect RNA from degradation by complexation and condensation, despite the RNA being exposed on the surface of the complex [17, 23]. Thus, we postulated that LNPs could be used to complex RNA in a similar manner to polyplexes by first forming the LNP and then electrostatically adsorbing the RNA to the surface of the particle. We further hypothesized that LNP formulations could be tailored and optimized to complex RNA by varying the properties of the complexing lipid.

Here, we systematically compare LNPs with saRNA on the interior versus exterior of the particle. We chose three complexing lipids based on their properties and previous use in mRNA formulations, including C12-200, an ionizable lipidoid previously used for siRNA and mRNA delivery [22, 24], dimethyldioctadecylammonium (DDA), a cationic lipid previously used in liposomal adjuvant formulations [25], and 1,2-dioleoyl-3-trimethylammonium propane (DOTAP), a cationic lipid previously used in mRNA LNP and emulsion formulations [5, 26]. We incorporated each lipid into LNP formulations based on previously used combinations of the complexing lipid, cholesterol, and a phospholipid [22], with saRNA either on the interior or exterior of the particle. We evaluated the in vitro transfection efficiency of each of the formulations, using firefly luciferase (fLuc) as a reporter protein and characterized how well each of the formulations protects saRNA from degradation by RNAse. Furthermore, we quantified the in vivo luciferase expression of each saRNA interior/exterior LNP formulations. Finally, we determined the immunogenicity of LNP formulations using an saRNA expressing HIV-1 Env gp140 as a model antigen.

## Materials and methods

### saRNA synthesis

saRNA encoding the nonstructural proteins of the Venezuelan equine encephalitis virus (VEEV) and either fLuc [27] or HIV-1 Env gp140 [28] was synthesized using in vitro transcription. Plasmid DNA (pDNA) was transformed into *Escherichia coli* and cultured in 50 mL of LB with 100 μg/mL carbenicillin (Sigma Aldrich, UK), and purified using a Plasmid Plus MaxiPrep kit (QIAGEN, UK). pDNA concentration and purity were measured on a NanoDrop One (Thermo Fisher, UK), and then linearized using MluI for 3 h at 37 °C, followed by heat inactivation at 80 °C for 20 min. Uncapped in vitro RNA transcripts were synthesized using 1 μg of linearized DNA template in a MEGAScript reaction (Promega, UK), according to the manufacturer’s protocol. Transcripts were then purified by overnight LiCl precipitation at −20 °C, pelleted by centrifugation at 14,000× RPM and 4 °C for 20 min, washed 1× with 70% EtOH, centrifuged at 14,000× RPM and 4 °C for 5 min, and then resuspended in UltraPure H_2_O. Purified transcripts were then capped by simultaneously using the ScriptCap™ m^7^G Capping System (CellScript, Madison, WI, USA) and ScriptCap™ 2′-O-Methyltransferase Kit (CellScript, Madison, WI, USA), according to the manufacturer’s protocol. Capped transcripts were then purified again by LiCl precipitation and stored at −80 °C.

### Production of LNPs with encapsulated saRNA

**Fig. 1:**
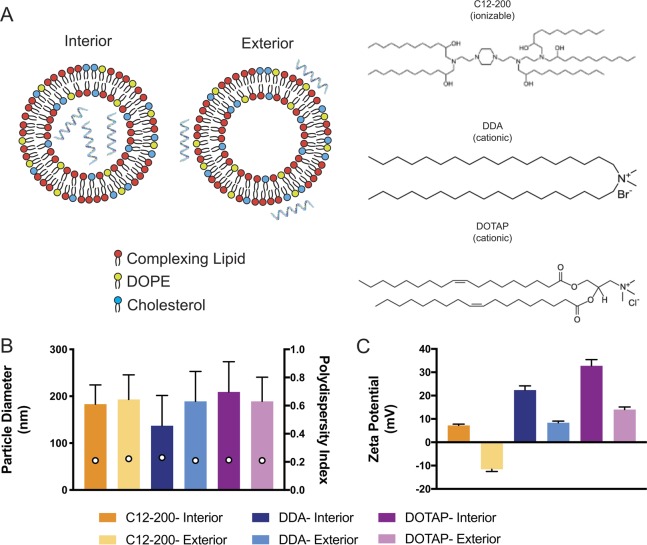
Characterization of saRNA lipid nanoparticle formulations. **a** Schematic of saRNA formulated on the interior or exterior of the lipid nanoparticles, with ionizable (C12-200) or cationic (DDA, DOTAP) complexing lipids. **b** Particle diameter (in nm) as determined by Nanoparticle tracking analysis (NTA) (bar graph) and their related polydispersity index (empty circles). **c** Surface charge of the LNPs as determined by zeta potential analysis measured on by the Zetasizer instrument. Bars represent means ± standard deviations for *n* = 3 for particle size and surface charge data

DDA bromide (Sigma, UK) and DOTAP (Avanti Polar Lipids, AL, USA) were used as received. C12-200 was synthesized by reacting 1 M equivalent of N^1^-(2-(4-(2-aminoethyl)piperazin-1-yl)ethyl)ethane-1,2-diamine (Enamine Ltd., Kyiv, Ukraine) with 7 M equivalents of 1,2-epoxydodecane (Sigma) at 80 °C for 2.5 days, according to published protocols [29]. LNPs with encapsulated saRNA (Fig. 1) were prepared on a μEncapsulator 1 System (Dolomite Bio, Royston, UK). The lipid solution was prepared by dissolving lipids in 90% EtOH at a total concentration of 1.5 mg/mL consisting of 35 mol% complexing lipid (C12-200, DDA, or DOTAP), 49 mol% cholesterol (Sigma), and 16 mol% 1,2-dioleoyl-sn-glycero-3-phosphoethanolamine (Avanti Polar Lipids). A volume of 100 μL of the lipid solution was loaded into one side of the μEncapsulator reservoir, while the other side was loaded with 100 μL of saRNA in citrate buffer (pH = 3), and the same solutions were loaded into the corresponding pumps. The ratio of complexing lipid to RNA was maintained at a N/P ratio of 12:1. A 50μm fluorophilic chip with a T-junction and subsequent phosphate buffer saline (PBS) (Sigma Aldrich, UK) dilution channel was used. LNPs were prepared using the following conditions: chip T = 70 °C, lipid solution pump pressure = 2000 Pa, citrate buffer pump pressure = 666 Pa, and PBS pump pressure = 2000 Pa. LNPs were purified by dialyzing against PBS in a 3500 MWCO dialysis cartridge (Thermo Fisher, UK) for 4 h.

### Production of LNPs with exteriorly complexed saRNA

LNPs with saRNA complexed to the exterior of the particle (Fig. 1) were prepared similarly to LNPs with encapsulated saRNA. However, instead of loading citrate buffer with saRNA into the reservoir, naive citrate buffer was loaded, and the instrument was run at identical conditions, until 5 mL of LNPs were produced. The LNPs were then purified by dialysis as stated above. For the final formulation, saRNA in citrate buffer (pH 3) was added at an N/P ratio of 12:1 and subsequently diluted in pH 7 PBS to the proper concentration 30–45 min prior to the start of the experiment. Exteriorly complexed LNPs were not further purified.

### Quantification of encapsulation efficiency

The saRNA loading in LNP formulations was quantified using a Quant-iT RiboGreen assay (Thermo Fisher, UK) as previously described [4]. Samples were diluted tenfold in 1× TE buffer containing 0.5% (v/v) Triton X-100 (Sigma Aldrich, UK). Standard solutions were also prepared in 1× TE containing 0.5% (v/v) Triton X-100 to account for any variation in fluorescence. The assay was performed according to the manufacturer’s protocol. Samples were loaded on a black, 96-well plate, and analyzed for fluorescence on a microplate reader (BMG LABTECH, UK) at an excitation of 485 nm and emission at 528 nm. In vitro and in vivo dosing was defined based on the calculated encapsulated dose.

### LNP characterization

The size and surface charge of LNPs were assessed with saRNA on either the interior or exterior of the particle. A volume of 100 μL of LNPs were diluted into 900 μL of PBS and equilibrated at room temperature (RT) (20 °C), prior to analysis. The particle size and polydispersity index (PDI) were characterized using a Zetasizer Nano ZS (Malvern Instruments, UK) with Zetasizer 7.1 software (Malvern, UK), using 850 μL of diluted particles in a 1-mL cuvette and the following settings: material refractive index of 1.4, absorbance of 0.01, dispersant viscosity of 0.882 cP, refractive index of 1.33, and dielectric constant of 79. Each sample was analyzed for up to 100 runs, or until measurement was suitably stabilized. Transmission electron microscopy (TEM) was performed on LNP formulations that were dialyzed against H_2_O overnight, stained with 2% uranyl acetate, and imaged on a TEM-2100 Plus Electron Microscopy (JEOL, Peabody, MA, USA) using a voltage of 80 kV.

### In vitro transfections

Transfections were performed in HEK293T.17 cells (ATCC, USA) that were maintained in complete Dulbecco’s Modified Eagle’s Medium (cDMEM) (Gibco, Thermo Fisher, UK) containing 10% fetal calf serum (FCS), 5 mg/mL l-glutamine, and 5 mg/mL penicillin/streptomycin (Thermo Fisher, UK). Cells were confirmed to be mycoplasma free prior to experimentation and were plated at a density of 50,000 cells per well in a clear 96-well plate, 48 h prior to transfection. A dose of 100 ng of interior or exterior complexed saRNA encoding fLuc was used per well in a volume of 100 μL of PBS, which was added to a well already containing 50 μL of transfection medium (DMEM with 5 mg/mL l-glutamine). For the “FCS” transfection condition, 50% (v/v) FCS was added to the transfection media immediately after formulation addition. For the “RNAse” condition, 3.8 milliarbitrary units (mAU) of RNAse (Life Technologies) per μg of RNA was added directly to the transfection media immediately after formulation addition. Cells were allowed to transfect for 4 h, and then the media was replaced with 100 μL of cDMEM. After 24 h from the initial transfection, 50 μL of media was removed from each well, and 50 μL of ONE-Glo™ D-luciferin substrate (Promega, UK) was added and mixed well. Then, the total volume of 100 μL was transferred to a white 96-well plate (Costar) and analyzed on a FLUOstar Omega plate reader (BMG LABTECH, UK), and background from to the media control wells subtracted.

### RNAse protection assay

In order to assess how well complexation on the interior/exterior of the LNP protected saRNA from degradation, samples were analyzed using an RNAse protection assay, similar to a method previously described [4]. A total of 3.8 mAU of RNAse (Life Technologies) per μg of RNA was added to the formulations and incubated for 30 min at room temperature. An identical formulation with no RNAse treatment was included as a negative control. RNAse was then inactivated using 6.4 mAU of proteinase K (New England Biolabs, UK) per μg RNA at 55 °C for 10 min. After inactivation, saRNA was extracted using a 1:1 (v/v) mixture of sample to 25:14:1 (v/v/v) phenol:chloroform:isoamyl alcohol. The extraction was performed by vortexing the solution well, and then centrifuging at 14,000× RPM for 10 min. The aqueous phase was removed by pipetting and mixed with NorthernMax Gly sample Loading Dye (Thermo Fisher, UK). The samples were then incubated at 75 °C for 10 min to denature the saRNA. A 1.2% agarose gel with 1× NorthernMax running buffer (Thermo Fisher) was prepared and allowed to completely cool before submerging in 1× NorthernMax running buffer. The samples were then added to the gel, and ran against Ambion Millenium RNA ladder (Thermo Fisher) at 120 V for 30 min. The gel was then imaged on a GelDoc-It^2^ (UVP, UK), and the intensity of the degraded and nondegraded bands was quantified using ImageJ (National Institutes of Health, USA). The % protection was defined as follows:

$${\mathrm{\% }}\,{\rm{Protection}} = 100 \ast \frac{{\rm{Intensity}}_{{\rm{RNAse}}\, {\rm{Treated}}\, {\rm{Sample}}}}{{\rm{Intensity}}_{{\rm{Naive}}\,{\rm{Sample}}}}$$

### In vivo luciferase imaging

Female BALB/c mice (Charles River, UK), 6–8 weeks of age, were placed into groups of *n* = 5 and housed in a fully acclimatized room. All animals were handled in accordance with the UK Home Office Animals Scientific Procedures Act of 1986 in accordance with an internal ethics board and a UK government-approved project and personal license. No randomization was used to determine how samples or animals were allocated to experimental groups. Researchers were blinded to group numbers while assessing the outcome by using generic group numbers. Group sizes were calculated to detect a difference of 1,000,000 relative light units (RLU) with a standard deviation of 200,000 RLU with a power of 0.9 and *α* = 0.05. Food and water were supplied ad libitum. Mice were injected intramuscularly (IM) in both hind leg quadriceps muscles with 5 μg of fLuc saRNA formulated either on the interior or exterior of C12-200, DDA, or DOTAP LNPs. After 7 days, the mice were injected intraperitoneally with 100 μL of XenoLight RediJect D-Luciferin Substrate (Perkin Elmer, UK) and allowed to rest for 10 min. Mice were then anesthetized using isoflurane and imaged on an In Vivo Imaging System FX Pro (Kodak Co., Rochester, NY, USA) equipped with Molecular Imaging Software Version 5.0 (Carestream Health, USA) for 10 min. A signal from each injection site was quantified using an equal detection area, using Molecular Imaging Software, and expressed as RLU.

### Immunogenicity study

Female BALB/c mice (Charles River, UK), 6–8 weeks of age, were placed into groups of *n* = 5. No randomization was used to determine how samples or animals were allocated to experimental groups. Researchers were blinded to group numbers while assessing the outcome by using generic group numbers. Group sizes were calculated to detect a difference of 200 ng/mL with a standard deviation of 40 ng/mL, with a power of 0.9 and *α* = 0.05. Mice were immunized IM in one hind leg quadriceps muscle with 5 μg of HIV-1 Env gp140-encoding saRNA formulated either on the interior or exterior of C12-200, DDA, or DOTAP LNPs to a total injection volume of 50 μL in 1× PBS and boosted with the identical formulation after 3 weeks. Tail bleeds were collected prior to each vaccination and 2 weeks after the boost injection. Blood was collected and centrifuged at 10,000× RPM for 5 min. The serum was harvested and stored at −20 °C.

### HIV-1 Env gp140-specific ELISA

A semiquantitative immunoglobulin IgG ELISA protocol was performed as previously described [30]. Briefly, 1 μg/mL of recombinant HIV-1 Env gp140 in PBS was coated onto ELISA plates, and standards were prepared by coating ELISA plate wells with anti-mouse Kappa (1:1000) and Lambda (1:1000) light chain (Southern Biotech, UK) in PBS. Plates were then blocked with 1% BSA/0.05% Tween-20 in PBS. After washing, diluted samples and purified IgG (Southern Biotech, UK) starting at 1000 ng/mL and titrating down the plate with fivefold dilution series were added to the plates, incubated for 1 h, and washed. A 1:2000 dilution of anti-mouse IgG-HRP (Southern Biotech, UK) was used for detection, and plates were developed using TMB (3,3′,5,5′-tetramethylbenidine), and the reaction was stopped after 5 min with Stop solution (Insight Biotechnologies, UK). Absorbance was read on a spectrophotometer (VersaMax, Molecular Devices) with SoftMax Pro GxP v5 software.

### Statistical analysis

Graphs and statistics were prepared in GraphPad Prism, version 7.0. Statistical differences were analyzed using a one-way ANOVA adjusted for multiple comparisons or a two-tailed, unpaired *t*-test with *α* = 0.05 used to indicate significance.

## Results

### Effect of interior/exterior saRNA complexation on LNP size, surface charge, and morphology

**Fig. 2:**
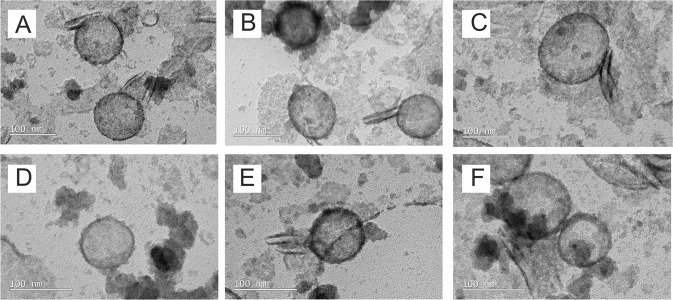
Transmission electron micrographs of LNP formulations. LNPs with saRNA on the interior (**a**–**c**) or exterior (**d**–**f**) of the particle, with C12-200 (**a**, **d**), DDA (**b**, **e**), and DOTAP (**c**, **f**) as the complexing lipid. Scale bar = 100 nm

After preparing the formulations with saRNA either on the interior or exterior of the particle (Fig. 1), we first sought to characterize how the different lipids, including C12-200 (ionizable), DDA (cationic), and DOTAP (cationic), affect the particle size and surface charge of these formulations (Fig. 1b, c). We observed that regardless of complexation to the interior or exterior of the LNPs, the average particle diameter was similar (~100–200 nm) (Fig. 1b, c). All LNPs were observed to have a similar PDI of ~0.2, indicating that there was a consistent range of particle sizes, irrespective of the arrangement of the saRNA. In addition, surface charge is an indicator of what is accessible at the surface of the particle, a negative charge indicating that the surface of the particle is mostly covered by RNA, while a positive charge indicates that the complexing lipids have not been saturated by RNA adsorption [31]. We found that the LNPs with encapsulated saRNA all had a positive surface, ranging from 8 to 30 mV, with DOTAP LNPs having the most positive surface charge (Fig. 1c). For the LNPs with saRNA adsorbed to the surface of the particle, both DDA and DOTAP had positive surface charges, ranging from 8 to 15 mV. In contrast, the C12-200 LNPs with saRNA on the exterior had a negative surface charge of −10 mV, thus indicating that the cations were quenched by the amount of RNA present in the formulation. The morphology was assessed using TEM, as shown in Fig. 2. The particles all exhibited a rounded morphology, with particle diameters equivalent to the NTA particle size characterization (~100–200 nm). Overall, we observed that the positioning of the saRNA on the interior or exterior of the LNPs has a limited effect on the size or PDI, and that for all the LNPs except the “C12-200 Exterior” condition, the exterior lipid was accessible and not saturated by saRNA.

### Effect of complexation position on transfection efficiency in the presence of FCS and RNAse

**Fig. 3:**
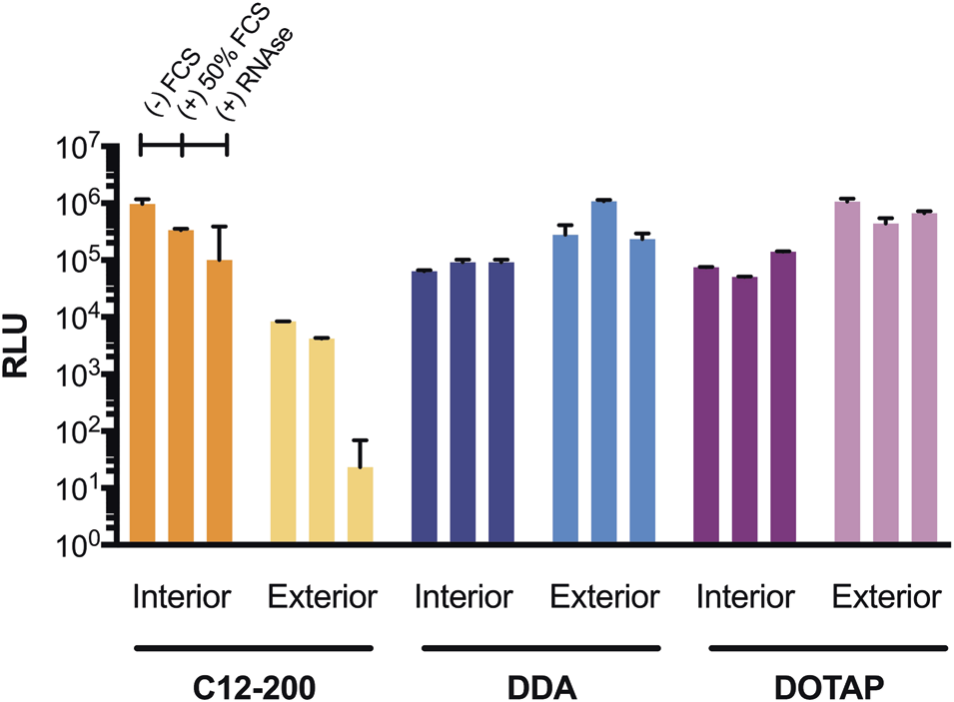
Transfection efficiency of fLuc-encoding saRNA delivered on the interior or exterior of LNPs. saRNA was transfected under standard conditions without FCS ((−) FCS), in the presence of FCS ((+) 50% FCS), or in the presence of exogenous RNAse ((+) RNAse). Luciferase expression was evaluated 24 h after transfection and is expressed as relative light units (RLU). Bars represent means ± standard deviations for *n* = 3

The formulations of fLuc-encoding saRNA were transfected into HEK293 cells, in order to determine the transfection efficiency of each formulation, and the role of the complexing lipid (Fig. 3). First, we tested the formulations under standard transfection conditions, which includes using a transfection media that does not contain FCS, which is known to bind to polyplexes and potentially decrease the transfection efficiency (Fig. 3). Based on the luciferase expression, we observed that the transfection efficiency of saRNA complexed to the exterior of C12-200 LNPs was ~2 orders of magnitude lower than when the saRNA was encapsulated within the C12-200 LNP (10^6^ RLU vs. 10^4^ RLU). However, the opposite trend was true for the cationic lipids, DDA and DOTAP, wherein complexing the saRNA to the exterior of the LNP resulted in higher transfection efficiency (~10^6^ RLU) compared with encapsulation within the particle (10^5^ RLU). Interestingly, including 50% FCS in the transfection, intended to mimic the high-protein conditions in vivo, did not inhibit the transfection efficiency, as we observed that all conditions had equivalent luciferase expression to the standard transfection conditions. Finally, we tested the formulations in a transfection, wherein exogenous RNAse was added in order to determine whether formulations subjected to a high amount of RNAse adequately protected the complexed RNA from degradation prior to cellular uptake. Remarkably, all the formulations besides the “C12-200 Exterior” LNPs did not exhibit decreased transfection efficiency in the presence of RNAse. Nevertheless, the transfection efficiency of the “C12-200 Exterior” LNPs decreased from 10^4^ RLU to 10 RLU. Overall, the C12-200 LNPs with encapsulated RNA and both the interior/exterior complexed DDA and DOTAP LNPs showed high transfection efficiency in vitro, with no loss of transfection in the presence of FCS and minimal degradation of the complexed saRNA prior to cellular uptake and expression.

### Interior/exterior complexation protect from RNAse degradation

**Fig. 4:**
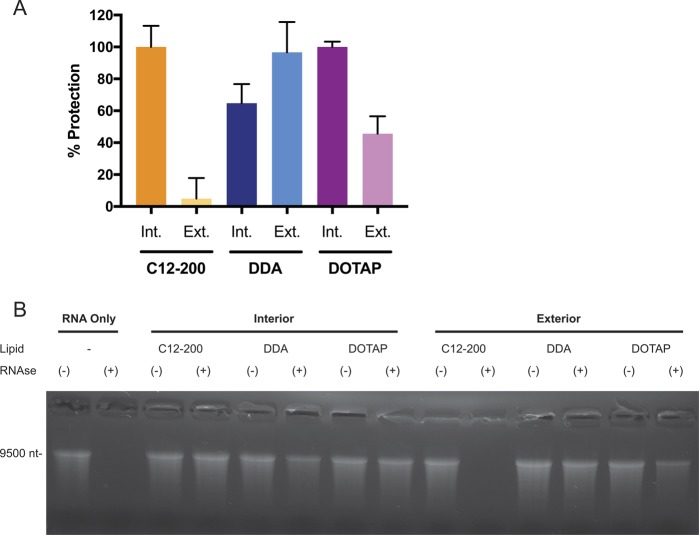
Effect of interior or exterior complexation on the protection of saRNA from RNAse degradation. **a** Formulations were subjected to RNAse, then purified by phenol–chloroform extraction, and analyzed by gel electrophoresis. Bars represent means ± standard deviations of the intensity of the nondegraded RNA band normalized to an equivalent sample that was not subjected to RNAse treatment for *n* = 2 samples. **b** Representative image of denaturing gel electrophoresis (Int: interior, Ext: exterior)

After observing that the transfection efficiency of exteriorly complexed saRNA on C12-200 LNPs, but not DDA or DOTAP LNPs, was inhibited by RNAse, we sought to characterize how well each of the formulations protected the saRNA from RNAse. Thus, we added RNAse directly to the formulation, and normalized the remaining amount of undegraded RNA to an equivalent sample that was not treated with RNAse (Fig. 4). We observed that the same amount of RNAse added to naked RNA completely degraded the RNA within 30 min. The C12-200 with encapsulated RNA protected the RNA almost 100%, while only ~10% of the RNA adsorbed to the outside of C12-200 LNPs was protected from degradation. These results mirror the decreased transfection efficiency of “C12-200 Exterior” LNPs in the presence of RNAse (Fig. 3). Similarly, for the DOTAP LNPs, almost 100% of the encapsulated saRNA was protected from RNAse degradation, whereas only ~45% of the exteriorly complexed saRNA remained intact after RNAse treatment. However, for the DDA LNPs, the opposite trend was observed, with only ~60% of the encapsulated saRNA protected from degradation, whereas ~95% of the saRNA adsorbed to the surface was protected. These results show that complexation to the surface of LNPs can be equally effective at protecting saRNA from degradation by RNAse as encapsulation within the LNP, but that it depends on the complexing lipid.

### In vivo LNP delivery of fLuc saRNA

**Fig. 5:**
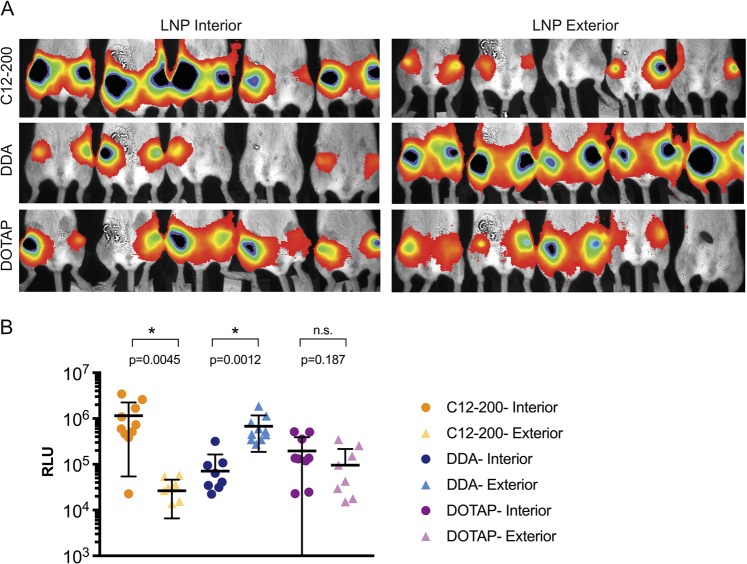
In vivo luciferase expression of saRNA complexed to the interior or exterior of LNPs. **a** In vivo imaging (IVIS) visualization of mice injected intramuscularly with 5 μg of fLuc saRNA per leg and imaged 7 days after injection. **b** Quantification of IVIS luciferase expression with a line at the mean ± standard deviation for *n* = 5 mice (*n* = 10 legs) per group. Units are expressed as relative light units (RLU). Asterisk indicates significance applying an unpaired *t*-test with *p < 0.05 (*p*-values indicated); n.s., nonsignificant

Given the high efficiency of our in vitro transfections, we wanted to characterize whether these formulations were capable of delivering saRNA in vivo, as it has been well established in the field that in vitro results are often non-predictive [32]. Female mice were injected with C12-200, DDA, and DOTAP formulations with saRNA on the interior or exterior of LNPs in both hind leg quadriceps muscles. Animals were then imaged for luciferase expression at day 7 (Fig. 5), as this was previously shown to be the peak luciferase expression for the VEEV replicon [33]. We observed that similarly to the in vitro transfection data (Fig. 3), the C12-200 LNPs with saRNA on the interior had significantly higher luciferase expression (~10^6^ RLU, *p* = 0.0045) than when saRNA was adsorbed to the surface (~10^4^ RLU). For the DDA LNPs, the opposite trend was observed; when saRNA was complexed to the exterior, the luciferase expression was significantly higher (~10^6^ RLU, *p* = 0.0012) than when encapsulated within the LNP (~10^5^ RLU). Interestingly, DOTAP showed no difference between interiorly and exteriorly complexed saRNA, as both conditions yielded a luciferase expression of ~10^5^ RLU (*p* = 0.187). The “C12-200 Interior” and “DDA Exterior” formulations had the highest overall luciferase expression (~10^6^ RLU) and were not significantly different from each other (*p* = 0.230). These data reinforce that saRNA can be efficiently delivered in vivo when either encapsulated in LNPs with an ionizable lipid or complexed to the surface of LNPs formulated with a cationic lipid. Moreover, we demonstrate that the identity of the cationic lipid used in the LNP formulation is an important factor that modulates delivery efficiency.

### Immunogenicity of interiorly or exteriorly complexed HIV-1 Env gp140-encoding saRNA

**Fig. 6:**
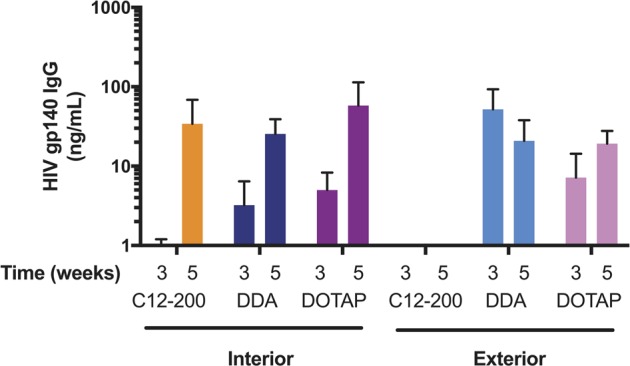
Antibody titers after immunization with HIV-1 Env gp140 saRNA complexed to the interior or exterior of LNPs. Bars represent means HIV-1 Env gp140-specific serum IgG antibody titer ± SEM, as determined by ELISA for *n* = 5 mice at each time point

After observing differential luciferase expression associated with the different LNP formulations in vivo, we sought to test whether these variations are reflected in the immunogenicity of saRNA vaccines. Female mice were injected with C12-200, DDA, and DOTAP LNP formulations with saRNA encoding HIV-1 Env gp140, as a model antigen, either on the interior or exterior of the particles. Mice received a prime injection, and then a boost after 3 weeks. HIV-1 Env gp140-specific IgG antibody titers were quantified at week 3, prior to the boost injection, and 2 weeks after the final injection at week 5 (Fig. 6). We observed similar IgG titers for each of the LNP formulations, with saRNA encapsulated within the particle (C12-200 In, DDA In, and DOTAP In), which increased by ~1 order of magnitude after a second injection. There was no HIV-1 Env gp140-specific IgG detected for the “C12-200 Exterior” formulation at either 3 or 5 weeks, which reflects the minimal in vivo luciferase expression we observed (Fig. 5). Interestingly, for both of the cationic LNP formulations (DDA Exterior and DOTAP Exterior), the IgG titers reached peak levels after a single injection. Despite trending differences between the formulations, none of the groups were statistically significantly different, as assessed by ANOVA adjusted for multiple comparisons. Overall, these results show that the formulations with encapsulated RNA induce equivalent IgG antibody responses against HIV-1 Env gp140, which were enhanced after a second injection, while the cationic formulations with exteriorly complexed saRNA achieved maximal IgG titers after a single injection.

## Discussion

Here, we show that LNPs formulated with cationic lipids and saRNA adsorbed to the surface efficiently deliver RNA in vitro and in vivo, with equivalent protein expression to LNPs formulated with an ionizable lipid and encapsulated RNA. Furthermore, despite RNA being exposed on the surface of the particles, we show that cationic lipids are able to complex, condense, and protect the RNA from RNAse degradation. In addition, we observed that cationic LNPs delivered saRNA in vivo, but that the composition of the cationic lipid is also a factor, as the delivery efficiency of DDA LNPs was significantly higher than DOTAP LNPs. Finally, both cationic LNPs with surface-adsorbed HIV-1 Env gp140 saRNA were shown to induce antibody responses that were equivalent to saRNA formulated on the interior of ionizable LNPs.

What are the potential benefits from complexing the saRNA on the surface, as opposed to encapsulating it within LNPs? One potential advantage is that a comprehensive quality control panel can be performed on a batch of LNPs, prior to the addition of RNA, which can then be incorporated at 100% efficiency. However, the main potential advantage is that this provides flexibility for complexing with different RNA constructs, as performed in these studies (Figs. 5 and 6). This approach could be particularly useful for emergency responses, wherein a batch of LNPs can be prepared in advance for immediate formulation at the onset of an outbreak. Even with sophisticated manufacturing instrumentation, production of LNPs at a laboratory scale can result in batch- to-batch variability, including disparate encapsulation efficiency, size, charge, and RNAse contamination. Excluding RNA from the initial production of particles enables easily reproducible batches of LNP formulations.

While the LNPs in these experiments all had similar particle diameters, ranging from 100 to 200 nm (Fig. 1), the “C12-200 Exterior” LNPs were the sole formulation with a negative surface charge. The formulation was complexed with RNA at pH 3, when the amine groups in the lipidoid are protonated [29], but then dialyzed in PBS for use in cell culture and animal studies. We postulate that the ionization of the lipid caused lower retention of the RNA, which then resulted in lower transfection and in vivo delivery efficiencies, as confirmed by the failure to induce an antibody response against HIV-1 Env gp140. Furthermore, despite saRNA complexation on the surface, the “C12-200 Exterior” LNPs were the only formulation that was susceptible to RNAse degradation during transfection (Fig. 3), although the “DDA Interior” and “DOTAP Exterior” did not completely protect saRNA from enzymatic degradation (Fig. 4). This suggests that while the “DDA Interior” LNPs were prepared with RNA on the interior of the particles, some of it is still present on the particle corona, although this does not seem to occur for the C12-200 or DOTAP “Interior” formulations. We observed that “DDA Exterior” and “DOTAP Exterior” LNPs still present a positive charge with an N/P ratio of 12:1, suggesting that a higher quantity of saRNA can be complexed to the surface of these particles. The “C12-200 Interior” and “DDA Exterior” formulations efficiently delivered saRNA in vivo (Fig. 5), in contrast to the “C12-200 Exterior” and “DDA Interior” LNPs, which were less effective delivery vehicles. This is evidenced by lower overall or no luciferase expression, which indicates that the saRNA in these cases is being taken up randomly, resulting in a weak signal that is amplified by the self-replicative properties of the RNA. Interestingly, the formulations with encapsulated HIV-1 Env gp140 saRNA all had equivalent serum antibody responses (Fig. 6), while the formulations with saRNA on the surface of cationic LNPs exhibited maximal antibody titer after a single immunization and did not require boosting. While the “C12-200 Interior” and the “DDA Exterior” LNPs had similar luciferase expression (Fig. 5), the “DDA Exterior” LNPs induced the highest antibody titers of the whole study after a single injection. This effect is likely due to adjuvanting properties of the DDA LNPs, which have previously been shown to act as adjuvants for protein vaccines [34, 35]. This was a relatively short vaccination schedule, and we suggest that utilizing a longer interval between vaccinations could further improve the antibody response, as this would provide more time for the protein expression to peak and completely dissipate before boosting [4, 36].

To our knowledge, this proof-of-concept study is the first systematic comparison of LNPs with saRNA on the interior or exterior of the particles. In order to be able to compare the formulations, we employed a single N/P ratio of 12:1, which can be further optimized for each formulation [37, 38]. It would be particularly useful to characterize the exact distribution of saRNA for each formulation, i.e., whether the saRNA is completely encapsulated or a portion is still accessible on the surface. The presented studies demonstrate the ability of LNPs to efficiently complex and deliver saRNA complexed on the surface of the particle, which presents an alternative approach to the paradigm of encapsulating RNA.
